# Supplementary material for: Design and Assembly of a Biofactory for (2S)-Naringenin Production in Escherichia coli: Effects of Oxygen Transfer on Yield and Gene Expression
Source: Biomolecules. 2023 Mar 20;13(3):565. doi: 10.3390/biom13030565 (PMC10046166; doi:10.3390/biom13030565)
Supplement: Supplementary file 1 [file biomolecules-13-00565-s001.zip › biomolecules-2223175-supplementary.pdf]

Table S1. Primers designed for the RT-qPCR.

Gene name	Forward (5′-3′)	Reverse (5′-3′)
fadE	GGTGAAGGGGCTGATTGATA	CAACTTTCCGCACTTTCTCC
fadB	CACTTCTTTAACCCGGTCCA	GTTAACCACAATCGGCGTCT
fadI	CGAAAGAATTAGGGCTGGTG	GCCAGACATCAATCGCAGTA
tesB	GCGGCCAGAGTGAAGATTTA	TTCAGGGACGGTCTCTTTTG
pyrD	CCACAGCCAGGTAATGACAA	AACCTTCGGCATCTACCAGA
frdA	AGCCGATCTTGCCATTGTAG	TATGGCTACGCATCGGGTAT
nuoA	TAGGCGGTTGGTTTTTAGGC	GATAAAACTTGGCGGACAGG
cyoD	CGTTCTGGATGGTGATGACA	CGTCATGTTCCAGCCTTCAT
mraY	GGGCTTCCTGTGGTTTAACA	ACATCGCCCATAAAGACCTG
murG	ATCTAATGGCTCAGGGTTGG	TTTGGCACTAAGTCCGCTTC
rrsA	GTGGGGAGCAAACAGGATTA	CCTCCAAGTCGACATCGTTT
pykF	GCCGTCTCGAGTTCAACAAT	ATTTACCGCCCTGAGTAGCA
lpdA	GAAGTTGACGACCGTGGTTT	TTGGTACGCAGCTGTTTGTC
accA	GGCTGAACGCTTTAAGATGC	GAGACATTTCACGCAGGTTG
gltA	GGGCAATCTTCATACCGTCA	TTCCGTCTTCCATGTTCACC
Pal	TAAAGACGACCCGCTGAACT	TCGTCCAGTTCAACAGCAAC
4cl	AGTTAACATCCAGCCGAACG	GTTTTCACCGTCAACCTGCT
Chs	ATGGGGTCAGCCGAAATCTA	TTTAACAGACGGACGCAGAC
chi	CGGTGCCACTGTTTTCTACA	TAAGCCCCAATACTCCATCG

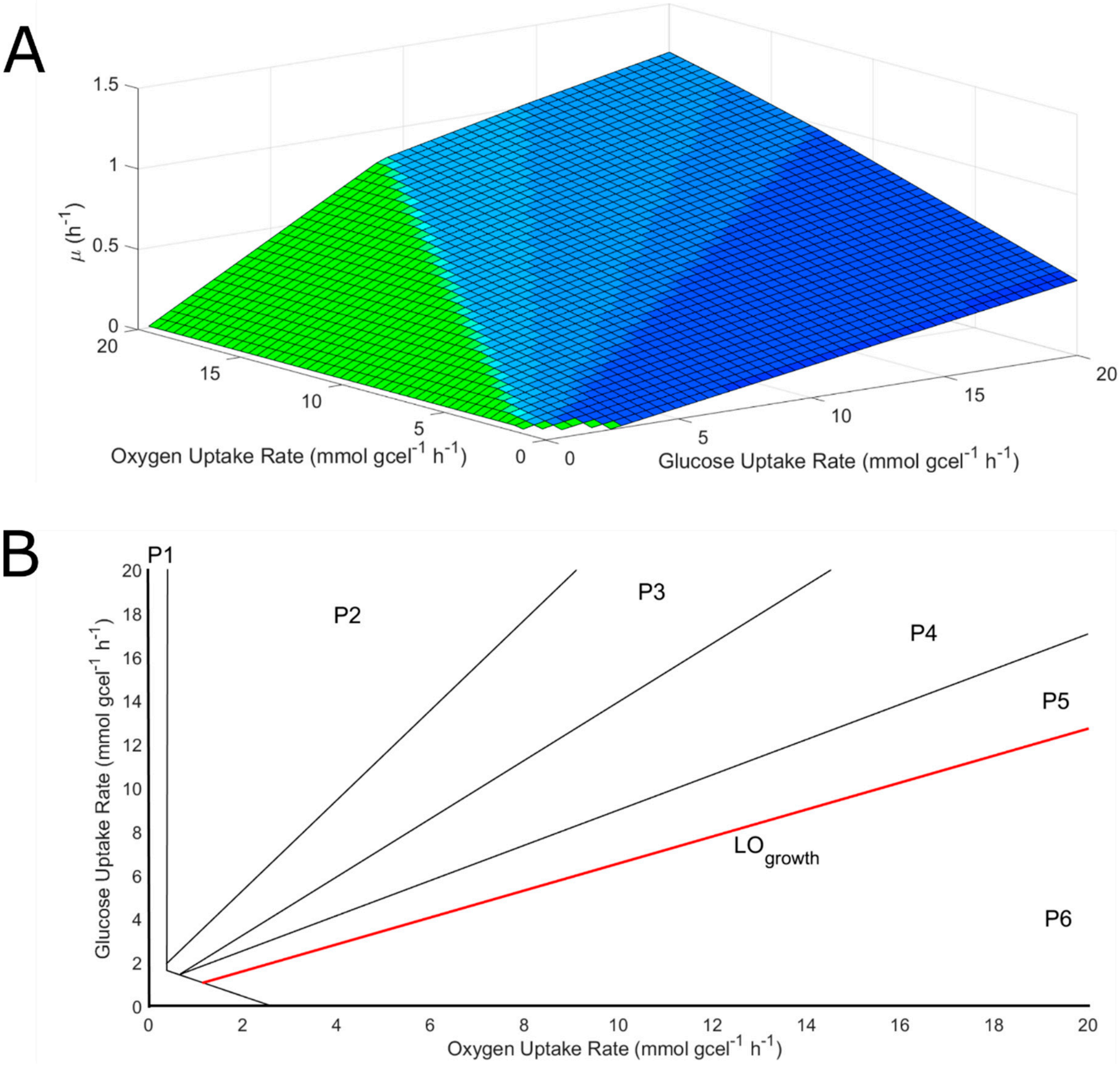

Figure S1. Phenotypic Phase Plane for *E. coli.* (A) The three-dimensional *E. coli* PhPP. The x-axis represents glucose uptake rate, the y-axis represents oxygen uptake rate, and the third dimension is the cellular growth rate. (B) A two-dimensional projection of the PhPP from the isoclines. The red line represents the optimal conditions for biomass yield. P_1-P_6 represent phases with different metabolic phenotypes.
